# Acute and long-term immune responses to SARS-CoV-2 infection in unvaccinated children and young adults with inborn errors of immunity

**DOI:** 10.3389/fimmu.2023.1084630

**Published:** 2023-01-20

**Authors:** Ana García-García, Claudia Fortuny, Victoria Fumadó, Iolanda Jordan, Laura Ruiz-López, Europa Azucena González-Navarro, Natalia Egri, Ana Esteve-Solé, Yiyi Luo, Alexandru Vlagea, Manel Monsonís Cabedo, Cristian Launes, Aleix Soler, Anna Codina, Manel Juan, Mariona Pascal, Angela Deyà-Martínez, Laia Alsina

**Affiliations:** ^1^ Study Group for Immune Dysfunction Diseases in Children (GEMDIP), Institut de Recerca Sant Joan de Déu, Barcelona, Spain; ^2^ Clinical Immunology and Primary Immunodeficiencies Unit, Pediatric Allergy and Clinical Immunology Department, Hospital Sant Joan de Déu, Barcelona, Spain; ^3^ Department of Surgery and Surgical Specializations, Facultat de Medicina i Ciències de la Salut, Universitat de Barcelona, Barcelona, Spain; ^4^ Clinical Immunology Program, Hospital Sant Joan de Déu-Hospital Clínic Barcelona, Barcelona, Spain; ^5^ Paediatric Infectious Diseases Unit, Hospital Sant Joan de Déu, Universitat de Barcelona, Barcelona, Spain; ^6^ Institut de Recerca Sant Joan de Déu, Barcelona, Spain; ^7^ CIBER of Epidemiology and Public Health, Madrid, Spain; ^8^ Translational Research Network in Paediatric Infectious Diseases (RITIP), Madrid, Spain; ^9^ Paediatric Intensive Care Unit, Hospital Sant Joan de Déu, Universitat de Barcelona, Barcelona, Spain; ^10^ Department of Immunology-CDB, Hospital Clínic-IDIBAPS, Universitat de Barcelona, Barcelona, Spain; ^11^ Department of Microbiology, Hospital Sant Joan de Déu, Esplugues de Llobregat, Barcelona, Spain; ^12^ Paediatrics Department, Hospital Sant Joan de Déu, Barcelona, Spain; ^13^ Paediatric Infectious Diseases Research Group, Institut de Recerca Sant Joan de Déu, Universitat de Barcelona, Barcelona, Spain; ^14^ Pathology Department and Biobank Department, Hospital Sant Joan de Deu, Esplugues de Llobregat, Barcelona, Spain; ^15^ Immunotherapy Platform, Hospital Sant Joan de Déu-Hospital Clínic, Universitat de Barcelona, Barcelona, Spain; ^16^ Spanish Network for Allergy - RETIC de Asma, Reacciones Adversas y Alérgicas (ARADYAL), Madrid, Spain

**Keywords:** SARS-CoV-2, COVID-19, primary immunodeficiency diseases, children, humoral immunity, cellular immunity

## Abstract

**Purpose:**

To describe SARS-CoV-2 infection outcome in unvaccinated children and young adults with inborn errors of immunity (IEI) and to compare their specific acute and long-term immune responses with a sex-, age-, and severity-matched healthy population (HC).

**Methods:**

Unvaccinated IEI patients up to 22 years old infected with SARS-CoV-2 were recruited along with a cohort of HC. SARS-CoV-2 serology and ELISpot were performed in the acute phase of infection (up to 6 weeks) and at 3, 6, 9, and 12 months.

**Results:**

Twenty-five IEI patients (median age 14.3 years, min.-max. range 4.5-22.8; 15/25 males; syndromic combined immunodeficiencies: 48.0%, antibody deficiencies: 16.0%) and 17 HC (median age 15.3 years, min.-max. range 5.4-20.0; 6/17 males, 35.3%) were included. Pneumonia occurred in 4/25 IEI patients. In the acute phase SARS-CoV-2 specific immunoglobulins were positive in all HC but in only half of IEI in whom it could be measured (n=17/25): IgG^+^ 58.8% (10/17) (p=0.009); IgM^+^ 41.2% (7/17)(p<0.001); IgA^+^ 52.9% (9/17)(p=0.003). Quantitative response (index) was also lower compared with HC: IgG IEI (3.1 ± 4.4) vs. HC (3.5 ± 1.5)(p=0.06); IgM IEI (1.9 ± 2.4) vs. HC (3.9 ± 2.4)(p=0.007); IgA IEI (3.3 ± 4.7) vs. HC (4.6 ± 2.5)(p=0.04). ELISpots positivity was qualitatively lower in IEI vs. HC (S-ELISpot IEI: 3/11, 27.3% vs. HC: 10/11, 90.9%; p=0.008; N-ELISpot IEI: 3/9, 33.3% vs. HC: 11/11, 100%; p=0.002) and also quantitatively lower (S-ELISpot IEI: mean index 3.2 ± 5.0 vs. HC 21.2 ± 17.0; p=0.001; N-ELISpot IEI: mean index 9.3 ± 16.6 vs. HC: 39.1 ± 23.7; p=0.004). As for long term response, SARS-CoV-2-IgM^+^ at 6 months was qualitatively lower in IEI(3/8, 37.5% vs. 9/10 HC: 90.0%; p=0.043), and quantitatively lower in all serologies IgG, M, and A (IEI n=9, 1.1 ± 0.9 vs. HC n=10, 2.1 ± 0.9, p=0.03; IEI n=9, 1.3 ± 1.5 vs. HC n=10, 2.9 ± 2.8, p=0.02; and IEI n=9, 0.6 ± 0.5 vs. HC n=10, 1.7 ± 0.8, p=0.002 –respectively) but there were no differences at remaining time points.

**Conclusions:**

Our IEI pediatric cohort had a higher COVID-19 pneumonia rate than the general age-range population, with lower humoral and cellular responses in the acute phase (even lower compared to the reported IEI serological response after SARS-CoV-2 vaccination), and weaker humoral responses at 6 months after infection compared with HC.

## Graphical Abstract

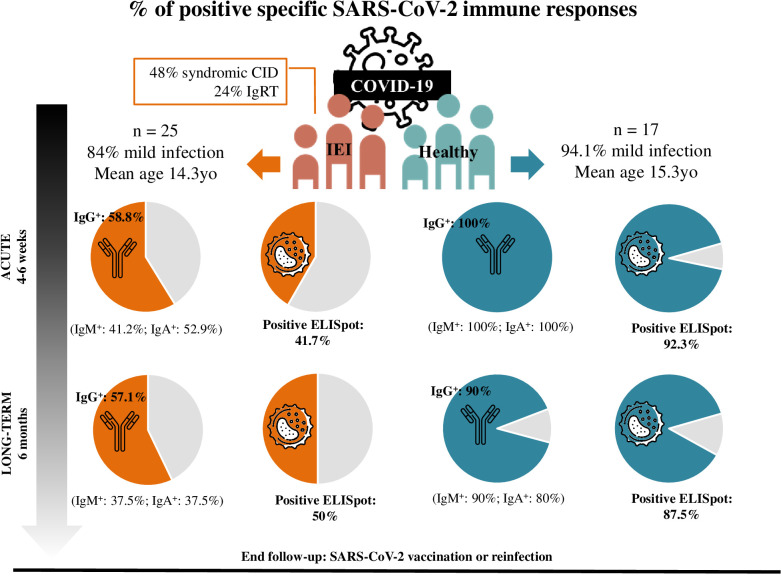

## 1 Introduction

Since the declaration of the COVID-19 pandemic in March 2020 (1), several efforts have been made to define the risk factors associated with SARS-CoV-2 infection and its severity. Older age remains the main risk factor identified for severe COVID-19 (2, 3); accordingly, the risk of hospitalization in children is low (4–7).

In healthy adult population there is generally a strong correlation between humoral and cellular responses against SARS-CoV-2 after natural infection: 96% of mild COVID-19 patients have positive SARS-CoV-2 S-protein–IgG until 8 to 12 months after infection, and memory CD4^+^ and CD8^+^ T-cells up to 12 months after infection (8). Healthy children in the acute phase have similar positive IgG antibody rates compared to adults, but with higher titer levels (9–11), as well as an antibody neutralizing capability similar to that of adults, up to 6-12 months after the infection (12). Nevertheless, other studies suggest children have lower seroconversion rates in the acute phase after infection (13, 14). Further, children develop a level of specific S-protein-T-cells twice that of adults, up to 6-12 months after infection, even when seronegative (12), whereas another group showed a low T-cell response rate at 6-7 months after the infection (not compared with adult population) (15).

Inborn errors of immunity (IEI) are a heterogeneous group of 485 disorders classified into 10 groups according to the immune components involved (16). A recent report of over 1330 patients with IEI reported so far shows that COVID-19 generally manifests clinically at a younger age, runs a more protracted course, and has a more severe outcome requiring hospitalization and/or ICU admission in many individuals with IEI compared to the epidemiology of SARS-CoV-2 infection in the general population (17). Although patients with IEI have had a heterogeneous clinical evolution during SARS-CoV-2 pandemics (18), in general terms COVID-19 has been less severe in children and young adults (19–21) compared to older IEI patients. The classical groups of IEI reported as affected with severe forms of SARS-CoV-2 infection have common variable immunodeficiency (CVID) with immune dysregulation (22–24) and combined immunodeficiency (25, 26), while surprisingly other IEI have been described as protective (24, 27). Furthermore, the pandemic has demonstrated that type I interferon (IFN-I) immunity is pivotal for the control of SARS-CoV-2 infection, through identification of anti-IFN-I–autoantibodies as well as inborn errors of IFN-I immunity. Autoantibodies against type-I IFN account for life-threatening COVID-19 in adults (2.6% of women, 12.5% of men) (28), and subjects younger than 40 years-old with positive anti-IFN-I autoantibodies have a COVID-19 index fatality rate increase of 0.84% (29). IEI such as *AIRE* deficiency confers a higher risk of critical COVID if there are pre-existing anti-INF-I autoantibodies (30). Inborn errors of IFN-I immunity account for about 10% of hospitalizations in children with COVID-19 pneumonia (31, 32), while X-linked TLR7 deficiency justifies 1% of critical pneumonia in men under 60 years old (33). Hence, IEI patients have been a focus of investigation to identify the pathways conveying risk for severe COVID-19.

Both humoral and cellular immune responses to COVID-19 (mainly S- and N- peptides) during acute and long-term infection have been studied in healthy population to guide SARS-CoV-2 preventive management (34–36). Currently, there is little knowledge regarding COVID-19 immune responses in IEI children compared to healthy population, with only a single study published to date (37). Moreover, there is great heterogeneity in study results since several factors influence immune responses, such as age (9–12, 14), sex (33), and COVID-19 severity (38–42), regardless of the type of IEI (37, 43). Studying COVID-19 immune responses in IEI provides not only insight into the vulnerability of reinfection in this population, but also reveals the immune mechanisms that are relevant for SARS-CoV-2 pathogenesis since IEI have dysfunctions in specific immunologic pathways (16), offering an excellent opportunity to develop therapeutic strategies (44).

The aim of this study was to describe COVID-19 clinical outcomes in unvaccinated children and young adults with IEI and their acute and long-term immune responses compared with a healthy control population.

## 2 Materials and methods

### 2.1 Study design and participants

A prospective non-matched case-control study was conducted in a single pediatric tertiary center that is an IEI referral center, in Barcelona, Spain. Patients aged 0 to 22 years old under follow up in the Unit due to IEI (according to the European Society for Immunodeficiencies Criteria Registry Working Party (45) were recruited when infected with SARS-CoV-2. A comparable age, sex, and infection severity-matched healthy population of children and young adults with COVID-19 was also recruited from the outpatient pediatric Infectious Disease clinic. None of the participants had previously been vaccinated against SARS-CoV-2, nor had they suffered a proven SARS-CoV-2 infection before the inclusion.

**Figure 1:**
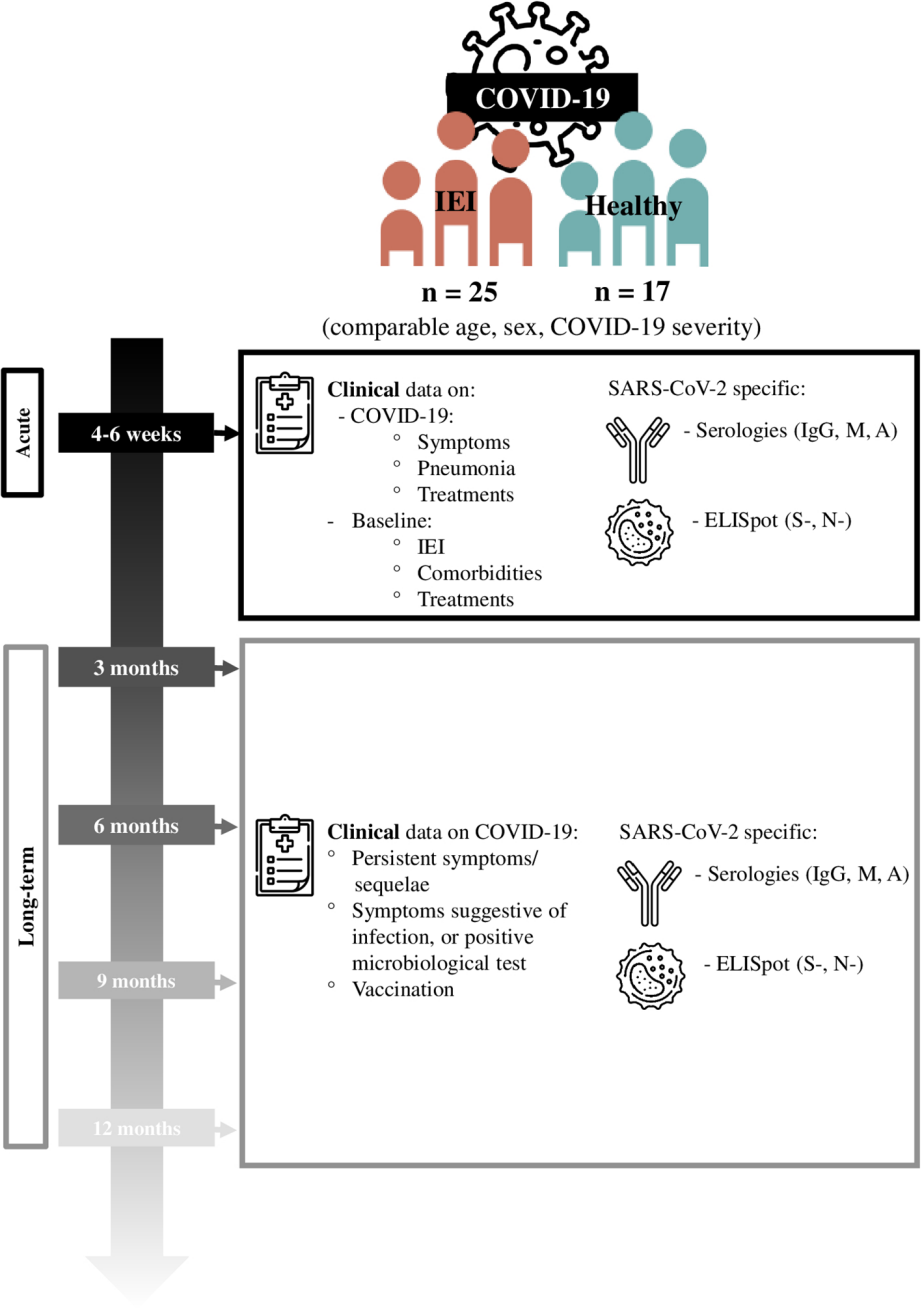
Study design.

Study design is detailed in **Figure 1**. Participants were recruited from May 2020 to January 2022 (21 months), and followed up during 1 to 12 months after the infection. During the follow-up, if the patient received the SARS-CoV-2 vaccination, suffered a second SARS-CoV-2 infection, or refused to continue with the study, the follow-up was interrupted.

All participants and/or their legal guardians signed the informed consent. This study was approved by the ethics committee of our institution (CEIm code PIC-60-20).

### 2.2 Definitions

#### 2.2.1 SARS-CoV-2 infection

SARS-CoV-2 infection was defined by positive specific nasopharyngeal and/or salivary antigen or PCR, and/or specific SARS-CoV-2 serology (IgG, IgM and/or IgA), according to confirmed case definition by the World Health Organization (WHO) and/or seroprevalence study (46, 47).

#### 2.2.2 COVID-19 severity

The COVID-19 clinical severity was based on the WHO criteria (48). Mild disease for children, adolescents, and adults is considered for those symptomatic without evidence of viral pneumonia or hypoxia; moderate disease in adolescents or adults for those with clinical signs of pneumonia (fever, cough, dyspnea, fast breathing) but not severe pneumonia (respiratory rate >30breaths/min, severe respiratory distress, or SpO_2_ <90% on room air); and moderate disease in children when they had clinical signs of non-severe pneumonia (cough or difficulty breathing and fast breathing and/or chest indrawing) but no signs of severe pneumonia (SpO_2_<90%, very severe chest wall indrawing, grunting, central cyanosis, or presence of any other general danger sign).

#### 2.2.2 Acute and long-term immune response

The acute immune response was assessed by testing the specific SARS-CoV-2 serologies and/or ELISpot up to 6 weeks after the patient had the first positive SARS-CoV-2 specific test (first proven SARS-CoV-2 infection). This cut-point for acute was based on the clinical criteria for post-acute COVID-19 syndrome, which is considered to begin 4 weeks after symptom onset (49).

For the long-term immune response study, serial clinical and analytical controls were performed every 3-6 months, up to 12 months after infection.

#### 2.2.3 Inborn error of immunity definition

A patient was considered to be affected by an IEI if he/she had a confirmed genetic diagnosis of IEI (16) or fulfilled the ESID (European Society for Immunodeficiencies) Registry diagnostic criteria (50). IEI groups were assigned, based on the International Union of Immunological Societies (IUIS), into 10 groups (16).

#### 2.2.4 Healthy control definition

Healthy controls were those without any of the following conditions: diagnosis of chromosomal disease, cardiac or midline malformations, oncological, hematological, or immune-related disease; any type of acute or chronic infection present at the time of extraction (other than first SARS-CoV-2 infection).

### 2.3 Clinical data

Data were collected from the medical chart regarding IEI type based on ESID (European Society for Immunodeficiencies) classification (50), baseline treatment, and demographic and general medical data for all participants. In addition, clinical data on SARS-CoV-2 infection were recorded prospectively for IEI patients and healthy controls (HC). Participants were asked about persistent SARS-CoV-2 symptoms/sequelae or newly confirmed SARS-CoV-2 infections or vaccination during the follow-up period.

### 2.4 Laboratory

Humoral response (serology) was assessed in frozen serum samples, and cellular response (ELISpot) was assessed in frozen peripheral blood mononuclear cells. Samples were collected in each follow-up visit, processed, and frozen in the following 6 hours at -80°C at the certified biobank of our institution, for further processing in batches to reduce variability.

#### 2.4.1 SARS-CoV-2 serologies

We used a serological assay based on the Luminex^®^ technique that has the benefit of a higher dynamic range compared to other assays, favoring the quantification of immunoglobulin levels. IgG, IgA, and IgM SARS-CoV-2-specific antibodies were determined by Luminex system against the receptor-binding domain of the spike glycoprotein. For 2 hours, 10 μL of serum was incubated with antigen-coupled beads at room temperature with agitation. After this, plates were washed 3 times and incubated with a biotinylated secondary antibody (IgM, IgA, or IgG; Sigma-Aldrich) for 45 minutes at room temperature with agitation. Plates were washed 3 times, and streptavidin-R-phycoerythrin (Sigma-Aldrich) was added for 30 minutes at room temperature with agitation. Plates were then washed 3 times and beads were re-suspended in phosphate-buffered saline (PBS). Plates were read using a Luminex xMAP 100 analyzer; positive values were assigned with Median Fluorescent Intensity (MFI) ratio 2 SD higher to a serum pool from pre-COVID pandemic samples; value ≥1 was considered to be positive. Sensitivity more than 10 days after onset of COVID-19 symptoms was 97% for IgG and 75% for IgM, and 100% specificity for both IgM and IgG (51, 52) The positive ratios (onwards “Index”: case or control sample MFI/pre-COVID sample MFI) were also considered for the analysis.

SARS-CoV-2 specific serologies were evaluated both in patients who were under immunoglobulin replacement therapy (IgRT) or not, given the absence of specific antibodies in the IgRT products administered at least before November 2020 (53), and also having described in other cohorts under IgRT that the measurement of these serologies is valid (54).

#### 2.4.2 ELISpot

SARS-CoV-2 specific T cell responses using IFN-γ ELISpot and flow cytometry: 2x10^5^ PBMCs or PBLs was stimulated with PepTivator^®^ SARS-CoV-2 Prot_S and N peptide pools (1 µg/mL, Miltenyi Biotec) in X-VIVO™ 15 medium supplemented with 10% heat-inactivated AB serum. Negative control wells lacked peptides, and positive control wells were incubated with phytohemaglutinin (PHA, 0.5 mg/mL). Cells were incubated for 72h at 37°C with 5% CO2 in pre-coated anti-IFN-γ MSIP white plates (mAb 1-D1K, Mabtech). Plates were then washed five times with PBS (Sigma-Aldrich) and incubated for 2h at room temperature with horseradish peroxidase (HRP)-conjugated anti-IFN-γ detection antibody (1 μg/mL; clone mAb-7B6-1; Mabtech). After a further five washes with PBS, tetramethylbenzidine (TMB) substrate was added, and spots were counted using the automated ELISpot Reader System (Autoimmun Diagnostika GmbH). PBMCs or PBLs after stimulation were stained for 20 min in the dark at room temperature with anti-CD3, CD4, and CD8 antibodies to determine T cell populations and antibodies against different activation markers—CD69, CD154, and CD137—and then analyzed on an Attune TM NxT cytometer. Data were analyzed with FlowJo (Tree Star Inc.). Antibodies were purchased from BD Biosciences-Pharmingen. Results were interpreted according to the manufacturer’s instructions.

### 2.5 Statistical analysis

Categorical and continuous variables were described as percentages and median values with ranges (min.-max.). For the comparative analysis, Chi-square test or Fisher exact test and Mann-Whitney *U* test were applied, as appropriate, to the data set. To objectify the differences of the variables measured in each patient at the different points of the follow-up, we used the lineal model (assumed sfericity or Greenhouse-Geisser). For data comparison Bonferroni adjustment was applied.

The analysis was performed using SPSS version 15.0 software (SPSS Inc., Chicago, IL), and statistical significance was set at p*-*value ≤ 0.05.

## 3 Results

### 3.1 Description of the IEI and HC cohort, and SARS-CoV-2 infection

A total of 25 IEI patients were included. Baseline characteristics and treatments of IEI patients are summarized in **Table 1**. The predominant IUIS group of IEI (16) was combined immunodeficiencies with syndromic features, representing 48%, followed by predominantly antibody deficiencies (16%). Six patients were under IgRT and 5 under chronic immunosupressants. Median age was 14.3 years old (mix-man: 4.5-22.8), with 15/25 males (60.0%). Although some patients had comorbidities associated with their IEI, only 4/25 (16.0%) patients had described risk factors associated with severe COVID-19 in children and young adults (55, 56): Patient 8 (P8) had Down syndrome, P10 had a cyanotic congenital heart disease, P12 had mild sleep apnea-hypopnea syndrome, and P24 had obesity (BMI >2SD WHO Growth reference median (57)).

**Table 1:** Baseline features of the IEI infected with SARS-CoV-2.

IEI (n=25)	
Age (years)^a^	14.3 (4.5-22.8)
Male	15/25 (60.0%)
Caucasian	20/25 (80.0%)
IEI	
Combined immunodeficiencies (CID)	3/25 (12.0%)
CID with associated syndromic features	12/25 (48.0%)
Predominantly antibody deficiencies	4/25 (16.0%)
Diseases of immune dysregulation	3/25 (12.0%)
Defects of phagocyte number, function, or both	1/25 (4.0%)
Defects of intrinsic and innate immunity	2/25 (8.0%)
Treatment	
Antibiotic, antiviral or antifungueal prophylaxis	7/25 (28.0%)
IgRT	6/25 (24.0%)
Immunomodulation therapy	5/25 (20.0%)
Corticosteroids	3/25 (12.0%)
Ruxolitinib	1/25 (4.0%)
Dupilumab	1/25 (4.0%)
COVID-19 severity	
Mild^b^	21/25 (92.0%)
Moderate^c^	3/25 (12.0%)
Severe	1/25 (4.0%)
Clinical symptoms	13/25 (52.0%)
Fever	7/25 (28.0%)
Cough	5/25 (20.0%)
Low-grade fever	3/25 (12.0%)
Odynophagia	3/25 (12.0%)
Runny nose	2/25 (8.0%)
Asthenia	2/25 (8.0%)
Smell and taste loss	2/25 (8.0%)
Headache	1/25 (4.0%)
Diarrhea	1/25 (4.0%)
Myalgia	1/25 (4.0%)
Ocular pain	1/25 (4.0%)
Pneumonia	4/25 (16.0%)
Hospitalization	4/25 (16.0%)
COVID-19 treatement	5/25 (20.0%)
Remdesivir	4/25 (16.0%)
Corticosteroids	2/25 (8.0%)
Antibiotic	4/25 (16.0%)
Heparin	1/25 (4.0%)
Conventional nasal prongs O_2_	1/25 (4.0%)
Comorbidities	
Respiratory disease	5/25 (20.0%)
Cardiovascular disease	3/25 (12.0%)
Neuropsychological disease	8/25 (32.0%)
Obesity	1/25 (4.0%)
Hemato-oncological disease	1/25 (4.0%)

a Age and follow-up are expressed as median (min.-max.).

b One mild case (asymptomatic, no pneumonia in the chest X-ray) was hospitalized because of the IEI risk (CID) and receive preventive remdesivir for 3 days.

c One moderate case (non-hypoxemic pneumonia) did not require hospital care (P4).

CID, combined immunodeficiency; O2, oxygen; IEI, inborn error of immunity; IgRT, immunoglobulin replacement treatment.

Seventeen HC were recruited. Comparing cases and controls, no differences in age (IEI: median 14.3 years-old, min.-max.: 4.5-22.8; controls: median 15.3, min.-max.: 4.5-22.8; p=0.5), gender (IEI: 15/25, 60.0% males; controls: 6/17,35.3% males; p=0.1), or severity (IEI: 3/25 hospitalized for pneumonia; controls: 1/17; OR: 2.2, 95% CI: 0.2-22.9; p=0.52) were found. No HC had risk factors for severe COVID-19 in children, as opposed to 4/25 in the IEI group (p=0.1).

Data on SARS-CoV-2 infection in the 25 patients are summarized in **Table 1** and **Supplementary Table 1.** Regarding COVID-19 severity, 21/25 (84.0%) IEI patients had mild infection (12/25, 48% were asymptomatic). With regards to the 4 patients with risk factors for severe COVID-19, only one (P24) had severe pneumonia, while the remaining cases were mild. Thus, only 4 patients had confirmed pneumonia (P1, P4, P24, P25) (16.0%). P1 was an 8-year-old boy with a combined immunodeficiency (Major Histocompatibility Complex Class II–MHCII- deficiency, OMIM 603200) who was under chronic corticosteroid treatment. He developed non-hypoxemic pneumonia, being treated early after first symptoms with remdesivir. His twin brother (P2), also affected with MHCII deficiency and under chronic corticosteroid treatment, was admitted for early remdesivir treatment when still asymptomatic, and remained without clinical or radiological signs of pneumonia). Patient (P4), a 13-year-old female with PGM3-deficiency (OMIM 172100), suffered non-hypoxemicpneumonia manifested with self-limited cough and low-grade fever. A routine thoracic scan was performed a week after this episode (bilateral diffuse ground-glass opacities suggestive of COVID-19 pneumonia, and a SARS-CoV-2 screening confirmed the recent infection: negative SARS-CoV-2 PCR, positive for specific IgA) (19). Lastly, a 19-year-old-patient with MyD88 deficiency (OMIM 602170) with obesity (P24) required conventional nasal prongs for 3 days, and his sister, 16 years old and affected by the same IEI (P25), had non-hypoxemic pneumonia (19). No patients with known IFN-I defects from our cohort were infected before vaccination.

When comparing pneumonia incidence in those with (1/6) and those without (3/19) IgRT (p=0.7), humoral (0/4) vs combined immunodeficiency (2/15) (p=0.6) and receiving immunosuppressive treatment (2/5) or not (2/20) (p=0.2), there were no significant differences.

Regarding COVID-19 sequelae in IEI patients, a 16-year-old female with an idiopathic CD4^+^ lymphopenia (P3) is under otorhinolaryngology rehabilitation after 2 years’ persistence of taste and smell disturbances leading to a significant dietary restriction. In addition, there was a patient with STAT1 gain-of-function (GOF) (P21, 15-years-old male) who developed an atypical Guillain-Barre 7 to 9 months after COVID-19 when re-exposed to SARS-CoV-2 infection (but without microbiological confirmation of infection). He fully recovered after immunomodulatory treatment. The remaining patients had no sequelae of SARS-CoV-2 infection.

Compared to the non-IEI cohort (**comparison detailed in** **Supplementary Table 2**), the severity and symptoms associated with COVID-19 were not significantly different, except for higher prevalence of headache in the non-IEI group (7/17, 41.2%) vs. IEI (1/25, 4.0%)(p=0.004; OR 0.06; 95% CI 0.006-0.5). Also, no patient in the control group reported long-term sequelae.

At least 2 individuals in the IEI cohort were reinfected before vaccination (P10 and P12: both were 6 years-old and had positive antigenic test, presenting mild COVID-19). No patient in the HC group was reinfected. Follow-up of both patients and controls was stopped as soon as they were reinfected or vaccinated.

### 3.2 Acute immune responses to SARS-CoV-2 infection

**Figure 2:**
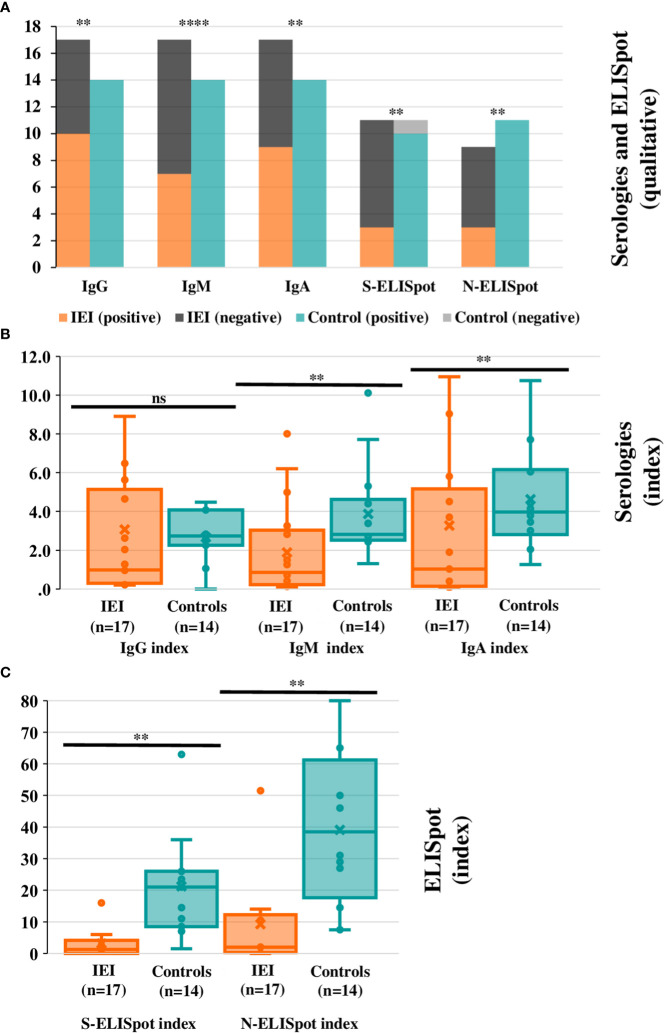
Humoral and cellular qualitative and quantitative responses in the acute phase post-SARS-CoV-2 infection. **(A)** Positive and negative SARS-CoV-2 specific IgG^+^, IgM^+^, IgA^+^, S-, and N-ELISpot are represented in the bars for IEI patients and controls. Panel **(B**, **C)** Index averages for SARS-CoV-2 specific IgG^+^, IgM^+^, IgA^+^ **(B)** S-, and N-ELISpot **(C)** are represented in the box-plot diagrams for IEI patients and controls. The box horizontal middle line represents the median, the box extremes represent lower quartile (Q1) and higher quartile (Q3); maximum and minimum values (without outliers) are represented by the horizontal lines in the extremes of the whiskers. The individual values between quartile 1 and 3 are represented by dots; the mean is represented by an “x” symbol. There are two extreme values that are not represented in the box-plot graphic: IgG index for cases: 16.7 (P9); IgA index for cases: 16.1 (P24). For all panels significance levels are marked as *p ≤ 0.05, **:p ≤ 0.01, ***p ≤ 0.001, ****p ≤ 0.0001, ns: p>0.05.

Regarding serological responses in the acute phase after infection in IEI patients compared with HC, IgG, IgM, and IgA were positive in all HC (100%, 14/14), but only positive in about half of IEI patients: IgG^+^ 58.8% (10/17) (p=0.009); IgM^+^ 41.2% (7/17) (p<0.001); IgA^+^ 52.9% (9/17) (p=0.003) (**Figure 2A**). In those patients with positive serology, the Ig index was higher in HC than in IEI patients: IgG index in IEI (3.1 ± 4.4) vs. HC (3.5 ± 1.5)(p=0.06); IgM index in IEI (1.9 ± 2.4) vs. HC (3.9 ± 2.4) (p=0.007); IgA index in IEI (3.3 ± 4.7) vs. HC (4.6 ± 2.5) (p=0.04) (**Figure 2B**).

As for the ELISpot, it could be performed in 13 HC, and only in 12/25 IEI patients. There were 13 patients in the IEI cohort that could not be analyzed for the following reasons: 3 patients were recruited after passing the acute-phase period, 4 patients had insufficient cells available after thawing to permit the technique to be used (all them combined immunodeficiencies), and for 6 patients the sample was not drawn due to technical reasons. Of the remaining 12 IEI patients, ELISpot was positive in 5 (41.7%), whereas in HC it was12/13 (92.3%) (p=0.01), and these differences remained significant if we separately compared positive specific S-ELISpot (IEI: 3/11, 27.3% vs. HC: 10/11, 90.9%; p=0.008) and positive specific N-ELISpot (IEI: 3/9, 33.3% vs. HC: 11/11, 100%; p=0.002). ELISpot index in IEI patients was significantly lower for both S-ELISpot (IEI: mean index 3.2 ± 5.0 vs. HC 21.2 ± 17.0; p=0.001) and N-ELISpot (IEI: mean index 9.3 ± 16.6 vs. HC: 39.1 ± 23.7; p=0.004) (**Figure 2C**).

### 3.3 Long-term immune responses to SARS-CoV-2 infection

**Figure 3:**
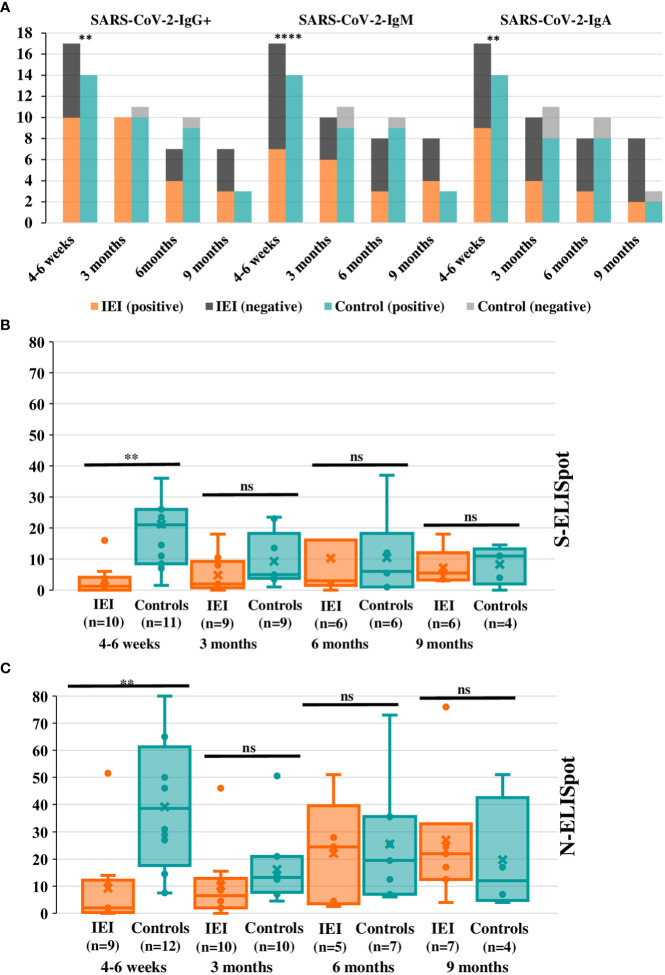
Positive serology and ELISpot indexes in cases and controls during the long-term phase post-SARS-CoV-2 infection. **(A)** Positive and negative SARS-CoV-2 specific IgG^+^, IgM^+^, and IgA^+^ are represented in the bars for IEI patients and controls. Only statistically significant differences are shown (*p ≤ 0.05, **p ≤ 0.01, ***p ≤ 0.001, ****p ≤ 0.0001). **(B)** S-ELISpot index comparison in cases-controls during the long-term period after the infection. There are two extreme values that are not represented in the box-plot graphic: ELISpot index for a control at 4-6 weeks: 63; ELISpot index at 6 months for P15: 48. **(C)** N-ELISpot index in cases and controls during the long-term follow-up. For **(B, C)**the box horizontal middle line represents the median, the extremes represent lower quartile (Q1) and higher quartile (Q3); maximum and minimum values (without outliers) are represented by the horizontal lines in the extremes of the whiskers. The individual values between quartile 1 and 3 are represented by dots; the mean is represented by an “x” symbol. For all panels significance levels are marked as *p ≤ 0.05, **p ≤ 0.01, ***p ≤ 0.001, ****p ≤ 0.0001, ns: p>0.05.

SARS-CoV-2-IgM^+^ at 6 months was lower in IEI compared to controls (3/8, 37.5%; 9/10: 90.0%; p=0.043), but these statistically significant differences were not maintained at 9 months (4/8, 50.0% vs. 3/3, 100%; p=0.2), nor were they present at 3 months (9/11, 81.8% vs. 6/10, 60%; p=0.4). SARS-CoV-2-IgG^+^ and SARS-CoV-2-IgA^+^ were not statistically different during the follow-up between the two groups (**Figure 3A**).

**Figure 4:**
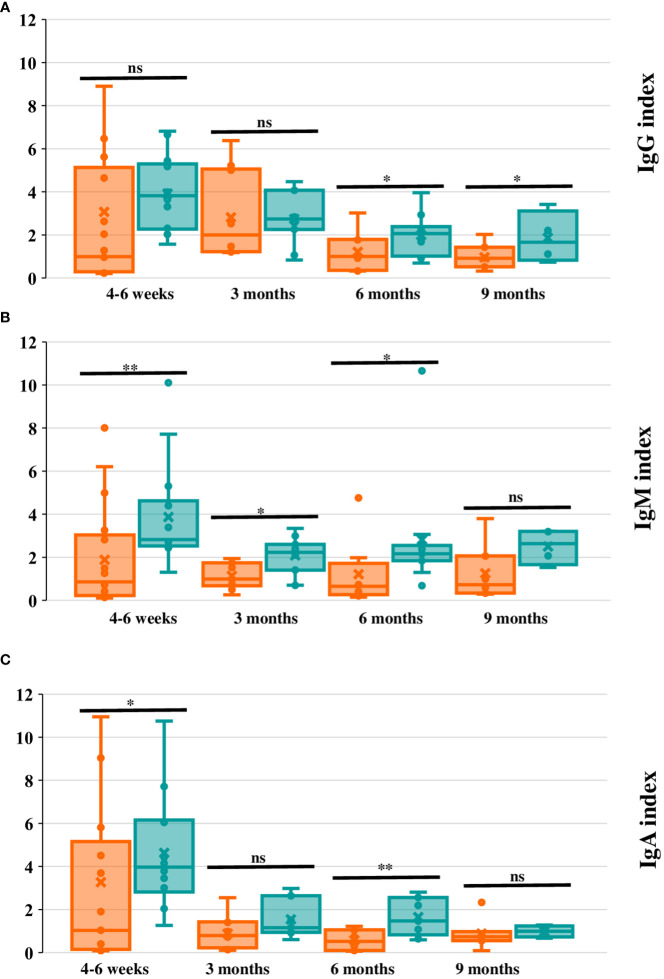
Ig index evolution in cases and controls during the long-term phase post-infection. **(A-C)**. IgG, IgM, and IgA index respectively (measured with Luminex assay) in cases (orange) and controls (green) at 4-6 weeks, and 3, 6 and 9 months after SARS-CoV-2 infection. The box horizontal middle line represents the median, the extremes represent lower quartile (Q1) and higher quartile (Q3); maximum and minimum values (without outliers) are represented by the horizontal lines in the extremes of the whiskers. The individual values between quartile 1 and 3 are represented by dots; the mean is represented by an “x” symbol. For all panels significance levels are marked as *p ≤0.05, **p ≤ 0.01, ***p ≤ 0.001, ****p ≤ 0.0001, ns: p>0.05. In **(A)** (IgG index) there is an extreme value that has not been represented at 4-6 weeks for P9, (IgG index 16.7). In **(C)** (IgA index) there is an extreme value that has not been represented at 4-6 months after the infection (P24, hypoxemic pneumonia; IgA index 16.1).

When the Ig index was compared between groups, SARS-CoV-2-IgG index was not different in IEI compared to HC at 3 months (IEI n=10, 2.8 ± 2.0 vs. controls n=11, 2.7 ± 1.2; p=0.8), but it was lower in IEI at 6 months post-infection (IEI n=9, 1.1 ± 0.9 vs controls n=10, 2.1 ± 0.9; p=0.03). There was also a lower SARS-CoV-2-IgM index in IEI at 3 months (IEI n=10, 1.1 ± 0. vs controls n=11, 2.1 ± 0.8; p=0.01) and 6 months (IEI n=9, 1.3 ± 1.5 vs controls n=10, 2.9 ± 2.8; p=0.02). For SARS-CoV-2-IgA index it was lower at 3 and 6 months in IEI, but this was only statistically significant at 6 months (IEI n=9, 0.6 ± 0.5 vs controls=10, 1.7 ± 0.8; p=0.002). At 9 months SARS-CoV-2-IgG index for the available IEI (n=8, 0.9 ± 0.6) was lower than that for controls (n=3, 2.2 ± 1.1; p=0.048). However, there were no statistically significant differences for IgM or IgA at 9 months. These data are represented in **Figure 4**.

**Figure 5:**
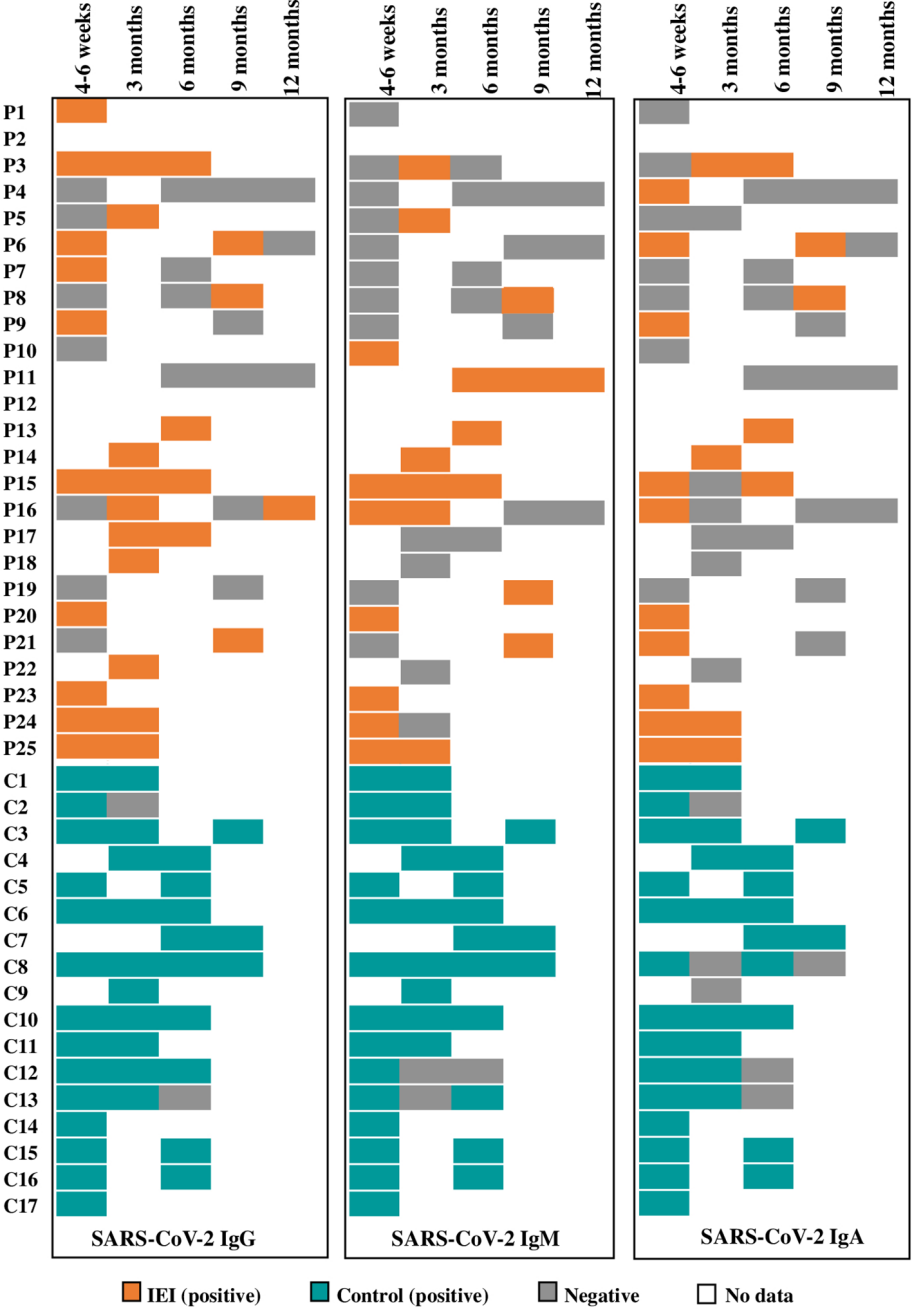
Patient by patient representation of SARS-CoV-2 positive and negative serologies during the follow-up.

Lastly, comparing the loss of the positive serology index from the first 6 weeks up to 6 months after the infection (not enough data were available to perform the statistical analysis at 9 months), in both cases and controls the trend of decrease of the IgG, IgM, and IgA index was significant from 4-6 weeks to 3 months and from 3 to 6 months after infection in each group. When comparing across groups, although the HC had a higher index than IEI, the intensity of the drop in the positive Ig index over time was not statistically different when comparing 4-6 weeks to 3 months and 3 to 6 months (**Figure 3A**). Nevertheless, qualitatively, we observed that the loss of positive serologies in the cases tended to occur earlier than in controls (**Figure 5**).

Cellular responses assessed by positive ELISpot were lower in IEI patients at 3 and 6 months when compared to HC (3 months: IEI 60.0% vs. HC 90.9% (p=0.2); 6 months: IEI 50.0% vs. HC 87.5% (p=0.2)), without reaching statistical significance, even when S- and N-ELISpot were analyzed separately. When comparing the index of decrease over time (both S- and N-ELISpot) there were no statistically significant differences across groups, at 3, 6, or 9 months after the infection. (**Figures 3B**, **C**). At 6 to 9 months’ follow-up, insufficient results were available to enable this comparison.

### 3.4 Immune responses to SARS-CoV-2 infection according to the type of IEI and IgRT

We then compared the immune responses of IEI patients who were with IgRT [n=6; median age 8.5 years old (min.-max.: 6.8-19.4)] and those without IgRT (n=19; median age 14.8 years old [min.-max.: 4.5-22.8)] (p=0.07). SARS-CoV-2-IgG^+^, -IgM^+^, and IgA^+^ at each analyzed time point after the infection showed no statistically significant differences between the two groups. IgG and IgM index at each time point (acute and long-term period) was higher for non-IgRT patients than IgRT, without reaching statistical significance. As for IgA index at 4-6 weeks after infection, non-IgRT patients had a higher index than patients on IgRT (n=14, average range 10.5 vs n=3, average range 2.0; p=0.008). As observed in IgG and IgM, IgA index during the long-term period was higher in non-IgRT, without reaching statistical significance. Regarding ELISpot positivity, there were no statistically significant differences between the two groups. Unlike serology index, S- and N-ELISpot index in patients under IgRT tended to have similar or even higher values compared to non-IgRT patients, without reaching statistical significance.

When comparing combined immunodeficiency (n=15; median age 11.6 years old; min.-max.: 4.5-19.4) and predominantly antibody immunodeficiency (n=4; median age 15.3 years old; min.-max.: 8.1- 7.7) (p=0.4), there were no statistically significant differences, either in humoral or in cellular immune responses in the acute or long-term period. Nor did comparison of the patients with (n=5) and those without immunosupressant treatment (n=25) show statistically significant differences in immune responses (data not shown).

## 4 Discussion

Currently, limited data on clinical evolution and specific immune responses to SARS-CoV-2 are available in pediatric IEI. Our results revealed that, from the clinical perspective, IEI have higher pneumonia incidence than age-matched general population. In the acute phase, both humoral and cellular immune responses were weaker compared to our healthy cohort. In the long-term, a trend to a greater decay in immune responses was observed in IEI patients compared to controls, but these differences did not reach statistical significance, probably due to the limited data available. IgRT had no impact on COVID-19 outcome or on immune responses.

Due to the fact that our HC were recruited intentionally with the same severity as IEI cases, we could not compare clinical outcomes between the two groups. Therefore, published series of COVID-19 in healthy children were used (7): our cohort of children and young adults with IEI had higher hospitalization and pneumonia rates (both: 4/25, 16.0%) compared to age-matched healthy population (both: 6.1%) (p=0.0496; OR 2.9 95% CI 1.0-8.5). However, pneumonia severity was comparable between the two groups (moderate pneumonia: IEI 8% vs non-IEI 5.3%, p=0.5; OR 0.6, 95% CI 0.1-2.7; severe and critical pneumonia: IEI 4% vs non-IEI 0.8%, p=0.12; OR 0.2, 95% CI 0.03-1.5). Our results are consistent with those reported by other comparable pediatric IEI cohorts such as Giardino et al.: 5.7% hospitalization rate, predominantly (26%) 22q11 deletion syndrome, but with a lower mortality rate than other pediatric IEI cohorts with predominantly SCID and primary hemophagocytic syndrome (25, 32, 58). Meyts et al. reported older IEI patients (predominantly antibody deficiency) that were more severe compared to our cohort (63% pneumonia, 33% hypoxemic pneumonia) (24), pointing up the influence of age and comorbidities as risk factors for severe COVID-19. As for acute symptoms reported by IEI patients compared to our HC with similar COVID-19 severity, there were no significant differences with the exception of greater incidence of headache in HC.

When analyzing the underlying IEI in the subgroup of patients suffering from COVID-19 pneumonia, we saw that two had a combined immunodeficiency (P1: MHCII-deficiency; P4: PGM3-deficiency), a form of IEI that has been reported as a risk factor for severe COVID- (24, 25, 27, 58). In addition, P4 was under dupilumab treatment, which has been described as protective for severe COVID-19 in non-IEI patients with atopic dermatitis (59). It may be, then, that dupilumab attenuated the natural course of COVID-19 disease in the patient, whose pneumonia was mild (ambulatory) and almost unnoticed. On the other hand, both P1 and his brother P2 were under long-term corticosteroid treatment, which may have increased the risk of severe COVID-19 (60): fortunately, they were treated early with remdesivir (61). Recent studies have shown that remdesivir administered within the first 7 days of SARS-CoV-2 infection provides higher odds for discharge and lower for intubation (61). Additionally, unexpectedly, a hypoxemic (P24) and non-hypoxemic pneumonia (P25) occurred in two siblings with MyD88-deficiency, an IEI that has not classically been associated with viral susceptibility (62).

Taste and smell disturbances are commonly described as COVID-19 post-acute sequelae (63) even in population under 21 years old, and these can last for 2 years after acute infection (64). In adults, this has been associated with chronic systemic conditions (65–67), and correlated with particular acute COVID-19 symptoms (68). Nevertheless, the precise etiopathogenesis of this disorder is still elusive (69, 70). Some IEI seem to be prone to long-term symptoms (71) such as XLA or other B-cell disorders, but the prevalence of taste and smell disturbances is not established in IEI. The patient with idiopathic CD4^+^ deficiency who presented chronic smell and taste disturbances (P3) developed a T-cell response (N-ELISpot) which remained positive with a high index value 6 months after acute infection. Lower inflammatory responses to SARS-CoV-2 infection have been related to dysgeusia and dysosmia (72), but no specific correlation with the characteristics of specific cellular responses has been reported. The post-COVID-19 Guillain-Barre case is more controversial. P21 (STAT1 GOF, 15 years-old) developed an atypical Guillain-Barre 9 months after testing positive for SARS-CoV-2. The impact of COVID-19 in Guillain-Barre incidence seems to be lower than with other infections and usually appears up to 2 months after acute infection (73–75). In our patient, all typical infectious triggers were ruled out (including herpes-virus), and no other trigger (including vaccination) could be identified. Thus, the role of SARS-CoV-2 infection in Guillain-Barre in this particular patient remains uncertain.

The comparisons between IEI subgroups (IgRT –which is associated to humoral immunity impairment-, immunosuppressive treatment, or cellular vs. humoral immunodeficiencies) did not show differences in the COVID-19 severity. These are factors commonly associated with worse outcomes (17, 24–26). The absence of detectable differences in our cohort could be ascribed to the limited samples size or to the young age of the patients, as being young age is a major protective factor for severe COVID-19 (2).

Regarding the acute immune response (up to 6 weeks after COVID-19), specific serologies (IgG, A, M) were qualitatively lower in IEI patients than in HC. For the quantitative results (index) IgG, IgM, and IgA were also lower in IEI, although no statistically significant differences for the IgG index were observed. This is not surprising since >50% of IEI patients in our cohort suffered from a humoral or combined underlying immunodeficiency. This may mean that the immediate protection against a reinfection is weaker in such patients with lower levels of seroprotection, not only for IgG but also for IgM, whose role in protecting against reinfection or cross-reactivity against other SARS-CoV-2 variants has been described (76). Nevertheless, and prior to vaccination, only 2 patients were reinfected. In contrast, positive seroconversion has been described in adult IEI patients with and without humoral defects after acute infection, with higher rates (86.7%) (77) vs. over 50% in our IEI patients. Compared to vaccine responses in IEI, specific serology positivity rates were lower in our IEI cohort after infection than those reported in IEI cohorts after 2 doses of RNA vaccine (78). This reinforces the advisability of vaccination in IEI patients, despite SARS-CoV-2 infection, although the role of humoral response is not fully defined for COVID-19 (79, 80), but seems important for certain IEI (e.g. CVID) (17).

Acute T-cell responses were also lower for S- and N-ELISpot in IEI patients, both quantitatively and qualitatively, compared to HC. This is consistent with another series that compared pediatric IEI response to non-IEI (37). Currently the clinical impact of these results is uncertain, and it is also unclear whether robust cellular responses can prevent severe disease progression during the acute phase of infection, or whether other factors may play a major role in this early phase (43, 81–86). Indeed, the two sicker patients in our cohort had a defect in innate immunity (MyD88 deficiency). These results point to the relevance of innate immunity in the acute control of SARS-CoV-2 infection (84).

Long-term immune responses in IEI were lower than in HC for specific serologies at 3 and 6 months after infection, which could suggests a major risk of reinfection (76) and reduced effectiveness of vaccines (35). Nevertheless, approximately 50% of the patients remained positive. This is consistent with other cohorts (adults with IEI) that have reported ‘high’ specific anti-spike antibody production (68.8%) at 1 to 7 months after SARS-CoV-2 infection (18), even in CVID patients.

As for T-cell responses, they were present in 50% and 87.5% of IEI patients at 6 and 9 months post-infection, respectively. At 6 months, they were lower compared with HC, without reaching statistical significance. Dowell et al. reported 84% of specific cellular responses at 6 months after infection in children (12), while 44% was reported by Kaaijk et al. at ten months after infection (84), and in 47.8% in ≥ 6 years old at 6-7 months after infection (12, 15). This last figure is rather similar to the values in our IEI cohort. Other series have consistently shown detectable T-cell responses in adult patients with primary and secondary antibody deficiency (81, 87–89). However, studies in larger cohorts of IEI patients are needed to learn about the duration and quality of the long-term immune responses, in order to calculate the reinfection risk and optimal vaccination schedule for these patients (34, 90).

The strengths of the study are: first, the sample size of pediatric IEI infected patients, taking into account the low incidence of IEI in the general population (16); second, the effort to recruit a matched healthy population for age, gender and severity of the disease, to enable immune responses comparisons; and third, the long follow-up period for the study of the long-term immune responses, which play a major role in vaccination programs. The limitations of the study are those inherent to sampling low volume of blood in pediatric patients and low cellularity in some IEI, increased by the use of frozen samples, all negatively impacting ELISpot results. Lastly, patients were lost during the follow-up because of reinfection or vaccination. In this regard, an asymptomatic reinfection could have occurred during follow-up impacting the positivization of serological and/or cellular results.

In conclusion, our pediatric and young adult cohort with IEI have a higher incidence of pneumonia compared to the general age-range population. Acute humoral and cellular responses against SARS-CoV-2 in IEI patients were qualitatively and quantitatively weaker compared to HC matched by age and severity. Our IEI cohort also had a lower humoral response after infection compared to the response reported in IEI patients after full SARS-CoV-2 vaccination (78). Long-term humoral and cellular responses were detectable in almost 50% of IEI patients at 6 months following infection, but specific IgG index at 6 months post-infection was decreased compared to HC.

## Data availability statement

The raw data supporting the conclusions of this article will be made available by the authors, without undue reservation.

## Ethics statement

The studies involving human participants were reviewed and approved by CEIm code PIC-60-20. Written informed consent to participate in this study was provided by the participants’ or their legal guardian/next of kin.

## Author contributions

AG-G, CF, IJ, MP, AD-M, and LA conceived and designed the study. AG-G, CF, VF, CL, AS, LR-L, and AD-M collected the data. Laboratory data analysis was performed by MM, NE, EG-N, LY, AV, AC, MJ, and MP. Statistical analysis was developed by AG-G and AD-M. The results were interpreted by AG-G, AV, LY, MJ, MP, AD-M, and LA. AG-G and AD-M drafted the article. Finally, AG-G, MP, AD-M, and LA discuss the data and made the critical revision of the article. All authors contributed to the article and approved the submitted version.

## Supplementary material

Supplementary Table 1 Patients with IEI baseline clinical characteristics and SARS-CoV-2 infection description.

Supplementary Table 2 Comparison of SARS-CoV-2 infection in IEI and non-IEI patients.

## References

[1] Cucinotta D Vanelli M. WHO declares COVID-19 a pandemic. Acta BioMed (2020) 91(1):157–60. doi: 10.23750/abm.v91i1.9397 PMC7569573 32191675

[2] O’Driscoll M Ribeiro Dos Santos G Wang L Cummings DAT Azman AS Paireau J. Age-specific mortality and immunity patterns of SARS-CoV-2. Nature (2021) 590(7844):140–5. doi: 10.1038/s41586-020-2918-0 33137809

[3] CDC. Risk for COVID-19 infection, hospitalization, and death by age group. Available at: https://www.cdc.gov/coronavirus/2019-ncov/covid-data/investigations-discovery/hospitalization-death-by-age.html.

[4] Ludvigsson JF. Systematic review of COVID-19 in children shows milder cases and a better prognosis than adults. Acta Paediatr (2020) 109(6):1088–95. doi: 10.1111/apa.15270 PMC7228328 32202343

[5] Docherty AB Harrison EM Green CA Hardwick HE Pius R Norman L. Features of 20 133 UK patients in hospital with covid-19 using the ISARIC WHO clinical characterisation protocol: Prospective observational cohort study. BMJ (2020) 369:m1985. doi: 10.1136/bmj.m1985 32444460 PMC7243036

[6] Castagnoli R Votto M Licari A Brambilla I Bruno R Perlini S. Severe acute respiratory syndrome coronavirus 2 (SARS-CoV-2) infection in children and adolescents: A systematic review. JAMA Pediatr (2020) 174(9):882–9. doi: 10.1001/jamapediatrics.2020.1467 32320004

[7] Martin B Dewitt PE Russell S Anand A Bradwell KR Bremer C. Characteristics, outcomes, and severity risk factors associated with SARS-CoV-2 infection among children in the US national COVID cohort collaborative. JAMA Netw Open (2022) 5(2). doi: 10.1001/jamanetworkopen.2021.43151 PMC8826172 35133437

[8] Zhang J Lin H Ye B Zhao M Zhan J Dong S. One-year sustained cellular and humoral immunities in coronavirus disease 2019 (COVID-19) convalescents. Clin Infect Dis (2022) 75(1):e1072–81. doi: 10.1093/cid/ciab884 PMC8524303 34609506

[9] Yang HS Costa V Racine-Brzostek SE Acker KP Yee J Chen Z. Association of age with SARS-CoV-2 antibody response. JAMA Netw Open (2021) 4(3):e214302. doi: 10.1001/jamanetworkopen.2021.4302 33749770 PMC7985726

[10] Breuer A Raphael A Stern H Odeh M Fiszlinski J Algur N. SARS-CoV-2 antibodies started to decline just four months after COVID-19 infection in a paediatric population. Acta Paediatr (2021) 110(11):3054. doi: 10.1111/apa.16031 34265136 PMC8444680

[11] Renk H Dulovic A Seidel A Becker M Fabricius D Zernickel M. Robust and durable serological response following pediatric SARS-CoV-2 infection. Nat Commun (2022) 13(1):1–11. doi: 10.1038/s41467-021-27595-9 35013206 PMC8748910

[12] Dowell AC Butler MS Jinks E Tut G Lancaster T Sylla P. Children develop robust and sustained cross-reactive spike-specific immune responses to SARS-CoV-2 infection. Nat Immunol (2021) 23(1):40–9. doi: 10.1101/2021.04.12.21255275 PMC8709786 34937928

[13] Toh ZQ Anderson J Mazarakis N Neeland M Higgins RA Rautenbacher K. Comparison of seroconversion in children and adults with mild COVID-19. JAMA Netw Open (2022) 5(3):e221313. doi: 10.1001/jamanetworkopen.2022.1313 35262717 PMC8908077

[14] Weisberg SP Connors TJ Zhu Y Baldwin MR Lin WH Wontakal S. Distinct antibody responses to SARS-CoV-2 in children and adults across the COVID-19 clinical spectrum. Nat Immunol (2021) 22(1):25–31. doi: 10.1038/s41590-020-00826-9 33154590 PMC8136619

[15] Ruedas-López A Berzosa-Sánchez A Illán-Ramos M Callejas-Caballero I Guillén-Martín S Bodas-Pinedo A. Longitudinal survey of humoral and cellular response to SARS-CoV-2 infection in children. Microbiol Res (2022) 264:127145. doi: 10.1016/j.micres.2022.127145 35973364 PMC9308145

[16] Tangye SG Al-Herz W Bousfiha A Cunningham-Rundles C Franco JL Holland SM. Human inborn errors of immunity: 2022 update on the classification from the international union of immunological societies expert committee. J Clin Immunol (2022) 1:1. doi: 10.1007/s10875-022-01289-3 PMC9244088 35748970

[17] Tangye SG. COVID Human Genetic Effort consortium. Impact of SARS-CoV-2 infection and COVID-19 on patients with inborn errors of immunity. J Allergy Clin Immunol (2022) S0091-6749(22)01568-8. doi: 10.1016/j.jaci.2022.11.010 PMC9746792 36522221

[18] Milota T Sobotkova M Smetanova J Bloomfield M Vydlakova J Chovancova Z. Risk factors for severe COVID-19 and hospital admission in patients with inborn errors of immunity - results from a multicenter nationwide study. Front Immunol (2022) 13:1. doi: 10.3389/fimmu.2022.835770 PMC8918471 35296097

[19] Deyà-Martínez A García-García A Gonzalez-Navarro EA Yiyi L Vlagea A Jordan I. COVID-19 in children and young adults with moderate/severe inborn errors of immunity in a high burden area in pre-vaccine era. Clin Immunol (2021) 230:108821. doi: 10.1016/j.clim.2021.108821 34391937 PMC8359496

[20] Marcus N Frizinsky S Hagin D Ovadia A Hanna S Farkash M. Minor clinical impact of COVID-19 pandemic on patients with primary immunodeficiency in Israel. Front Immunol (2021) 11. doi: 10.3389/fimmu.2020.614086 PMC7840610 33519822

[21] Milito C Lougaris V Giardino G Punziano A Vultaggio A Carrabba M. Clinical outcome, incidence, and SARS-CoV-2 infection-fatality rates in Italian patients with inborn errors of immunity. J Allergy Clin Immunol Pract (2021) 9(7):2904–2906.e2. doi: 10.1016/j.jaip.2021.04.017 33894392 PMC8059325

[22] Shields AM Burns SO Savic S Richter AG. COVID-19 in patients with primary and secondary immunodeficiency: The united kingdom experience. J Allergy Clin Immunol (2021) 147(3):870–875.e1. doi: 10.1016/j.jaci.2020.12.620 PMC7737531 33338534

[23] Shields AM Anantharachagan A Arumugakani G Baker K Bahal S Baxendale H. Outcomes following SARS-CoV-2 infection in patients with primary and secondary immunodeficiency in the UK. Clin Exp Immunol (2022) 209:247–58. doi: 10.1093/cei/uxac008 PMC8807296 35641155

[24] Meyts I Bucciol G Quinti I Neven B Fischer A Seoane E. Coronavirus disease 2019 in patients with inborn errors of immunity: An international study. J Allergy Clin Immunol (2021) 147(2):520–31. doi: 10.1016/j.jaci.2020.09.010 PMC7832563 32980424

[25] Delavari S Abolhassani H Abolnezhadian F Babaha F Iranparast S Ahanchian H. Impact of SARS-CoV-2 pandemic on patients with primary immunodeficiency. J Clin Immunol (2021) 41(2):345–55. doi: 10.1007/s10875-020-00928-x PMC7707812 33263173

[26] Babaei M Kanannejad Z Sepahi N Alyasin S. The effect of COVID-19 pandemic on patients with primary immunodeficiency: A cohort study. Iran J Med Sci (2022) 47(2):162–6. doi: 10.1007/s10875-020-00928-x PMC8919309 35291437

[27] Bucciol G Tangye SG Meyts I. Coronavirus disease 2019 in patients with inborn errors of immunity: Lessons learned. Curr Opin Pediatr (2021) 33(6):648. doi: 10.1097/MOP.0000000000001062 34734915 PMC8577305

[28] Bastard P Rosen LB Zhang Q Michailidis E Hoffmann HH Zhang Y. Autoantibodies against type I IFNs in patients with life-threatening COVID-19. Science (80-) (2020) 370(6515):eabd4585. doi: 10.1126/science.abd4585 PMC7857397 32972996

[29] Manry J Bastard P Gervais A Le Voyer T Rosain J Philippot Q. The risk of COVID-19 death is much greater and age-dependent with type I IFN autoantibodies. Res Sq (2022) 119(21):e2200413119. doi: 10.1073/pnas.2200413119 PMC9173764 35576468

[30] Bastard P Orlova E Sozaeva L Lévy R James A Schmitt MM. Preexisting autoantibodies to type I IFNs underlie critical COVID-19 pneumonia in patients with APS-1. J Exp Med (2021) 5:29. doi: 10.1084/jem.20210554 PMC8077172 33890986

[31] Zhang Q Matuozzo D Le Pen J Lee D Moens L Asano T. Recessive inborn errors of type I IFN immunity in children with COVID-19 pneumonia. J Exp Med (2022) 219(8):e20220131. doi: 10.1084/jem.20220131 35708626 PMC9206114

[32] Abolhassani H Delavari S Landegren N Shokri S Bastard P Du L. Genetic and immunological evaluation of children with inborn errors of immunity and severe or critical COVID-19. J Allergy Clin Immunol (2022) 150(5):1059–73. doi: 10.1016/j.jaci.2022.09.005 PMC9472457 36113674

[33] Asano T Boisson B Onodi F Matuozzo D Moncada-Velez M Renkilaraj MRLM. X-Linked recessive TLR7 deficiency in $~$1% of men under 60 years old with life-threatening COVID-19. Sci Immunol (2021) 6(62):65. doi: 10.1126/sciimmunol.abl4348 PMC8532080 34413140

[34] Safont G Latorre I Villar-Hernández R Stojanovic Z Marín A Pérez-Cano C. Measuring T-cell responses against SARS-CoV-2 is of utility for disease and vaccination management. J Clin Med (2022) 11(17):5103. doi: 10.3390/jcm11175103 36079033 PMC9457376

[35] Mantus G Nyhoff LE Edara VV Zarnitsyna VI Ciric CR Flowers MW. Pre-existing SARS-CoV-2 immunity influences potency, breadth, and durability of the humoral response to SARS-CoV-2 vaccination. Cell Rep Med (2022) 3(4):100603. doi: 10.1016/j.xcrm.2022.100603 35480625 PMC8960152

[36] Wirsching S Harder L Heymanns M Gröndahl B Hilbert K Kowalzik F. Long-term, CD4 + memory T cell response to SARS-CoV-2. Front Immunol (2022) 13:800070. doi: 10.3389/fimmu.2022.800070 35514974 PMC9065554

[37] Awuah A Zamani A Tahami F Davis M Grandjean L Buckland M. T Cell responses to SARS-CoV-2 in healthy controls and primary immunodeficiency patients. Clin Exp Immunol (2022) 207(3):uxac001–uxac001. doi: 10.1093/cei/uxac001 35020892 PMC8807284

[38] Menges D Zens KD Ballouz T Caduff N Llanas-Cornejo D Aschmann HE. Heterogenous humoral and cellular immune responses with distinct trajectories post-SARS-CoV-2 infection in a population-based cohort. Nat Commun (2022) 13(1). doi: 10.1038/s41467-022-32573-w PMC9386650 35982045

[39] Mazzoni A Maggi L Capone M Vanni A Spinicci M Salvati L. Heterogeneous magnitude of immunological memory to SARS-CoV-2 in recovered individuals. Clin Transl Immunol (2021) 10(5):e1281. doi: 10.1002/cti2.1281 PMC8101693 33976879

[40] Rydyznski Moderbacher C Ramirez SI Dan JM Grifoni A Hastie KM Weiskopf D. Antigen-specific adaptive immunity to SARS-CoV-2 in acute COVID-19 and associations with age and disease severity. Cell (2020) 183(4):996–1012.e19. doi: 10.1016/j.cell.2020.09.038 PMC7494270 33010815

[41] Moss P. The T cell immune response against SARS-CoV-2. Nat Immunol (2022) 23(2):186–93. doi: 10.1038/s41590-021-01122-w 35105982

[42] Demaret J Lef evre G Vuotto F Trauet J Duhamel A Labreuche J. Severe SARS-CoV-2 patients develop a higher specific T-cell response. Clin Transl Immunol (2020) 9. doi: 10.1002/cti2.1217 PMC7757425 33376594

[43] Giardino G Romano R Coppola E Cillo F Borzachiello C De Luca M. SARS-CoV-2 infection in the immunodeficient host: Necessary and dispensable immune pathways. J Allergy Clin Immunol Pract (2021) 9(9):3237–3248. doi: 10.1016/j.jaip.2021.06.045 PMC8279920 34273582

[44] Tangye SG Bucciol G Meyts I. Mechanisms underlying host defense and disease pathology in response to severe acute respiratory syndrome (SARS)-CoV2 infection: Insights from inborn errors of immunity. Curr Opin Allergy Clin Immunol (2021) 21(6):515–24. doi: 10.1097/ACI.0000000000000786 34494617

[45] Seidel MG Kindle G Gathmann B Quinti I Buckland M van Montfrans J. The European society for immunodeficiencies (ESID) registry working definitions for the clinical diagnosis of inborn errors of immunity. J Allergy Clin Immunol Pract (2019) 7(6):1763–70. doi: 10.1016/j.jaip.2019.02.004 30776527

[46] WHO COVID-19 case definition. Available at: https://www.who.int/publications/i/item/WHO-2019-nCoV-Surveillance_Case_Definition-2022.1.

[47] Kundu D Gautam P Dayanand D Gunasekaran K Manesh A Sebastian M. The role and diagnostic accuracy of serology for COVID-19. BMC Infect Dis 22(1):1–8. doi: 10.1186/s12879-022-07361-y PMC9017961 35439957

[48] Health Organization W. Guideline clinical management of COVID-19 patients: living guideline, Vol. 18. (2021).

[49] Nalbandian A Sehgal K Gupta A Madhavan MV McGroder C Stevens JS. Post-acute COVID-19 syndrome. Nat Med (2021) 27(4):601–15. doi: 10.1038/s41591-021-01283-z PMC8893149 33753937

[50] European Society for Immunodeficiencies. Available at: https://esid.org/Working-Parties/Registry-Working-Party/Diagnosis-criteria.

[51] Cucchiari D Egri N Bodro M Herrera S Del Risco-Zevallos J Casals-Urquiza J. Cellular and humoral response after MRNA-1273 SARS-CoV-2 vaccine in kidney transplant recipients. Am J Transplant 21(8):2727. doi: 10.1111/ajt.16701 PMC8222867 34036720

[52] Hoffman T Kolstad L Lindahl JF Albinsson B Bergqvist A Rönnberg B. Diagnostic potential of a luminex-based coronavirus disease 2019 suspension immunoassay (COVID-19 SIA) for the detection of antibodies against SARS-CoV-2. Viruses (2021) 13(6):993. doi: 10.3390/v13060993 34073484 PMC8227055

[53] Romero C Díez JM Gajardo R. Anti-SARS-CoV-2 antibodies in healthy donor plasma pools and IVIG products. Lancet Infect Dis (2021) 21(6):765–6. doi: 10.1016/S1473-3099(21)00059-1 PMC7906732 33606999

[54] Antolí A Rocamora-Blanch G Framil M Mas-Bosch V Navarro S Bermudez C. Evaluation of humoral and cellular immune responses to the SARS-CoV-2 vaccine in patients with common variable immunodeficiency phenotype and patient receiving b-cell depletion therapy. Front Immunol (2022) 13(April):1–12. doi: 10.3389/fimmu.2022.895209 PMC9098934 35572562

[55] Woodruff RC Campbell AP Taylor CA Chai SJ Kawasaki B Meek J. Risk factors for severe COVID-19 in children. Pediatrics (2022) 149(1). doi: 10.1542/peds.2021-053418 PMC9213563 34935038

[56] Schober T Caya Mscph C Md MB Bayliss A Bitnun A Bowes Msc J. Risk factors for severe PCR-positive SARS-CoV-2 infection in hospitalized children: a multicenter cohort study. BMJ Paediatr Open (2022) 6(1):e001440. doi: 10.1136/bmjpo-2022-001440 PMC9358955 36053578

[57] Obesity and overweight . Available at: https://www.who.int/news-room/fact-sheets/detail/obesity-and-overweight.

[58] Karakoc Aydiner E Bilgic Eltan S Babayeva R Aydiner O Kepenekli E Kolukisa B. Adverse COVID-19 outcomes in immune deficiencies: Inequality exists between subclasses. Allergy (2022) 77(1):282–95. doi: 10.1111/all.15025 PMC8441734 34314546

[59] Ungar B Glickman JW Golant AK Dubin C Marushchak O Gontzes A. COVID-19 symptoms are attenuated in moderate-to-Severe atopic dermatitis patients treated with dupilumab. J Allergy Clin Immunol Pract (2022) 10(1):134. doi: 10.1016/j.jaip.2021.10.050 34737108 PMC8558098

[60] Lafaurie M Martin-Blondel G Delobel P Kamar N Charpentier S Sommet A. Impact of previous exposure to systemic corticosteroids on unfavorable outcome in patients hospitalized for COVID-19. BMC Pharmacol Toxicol (2021) 22(1):1–6. doi: 10.1186/s40360-021-00480-3 33706794 PMC7948656

[61] Jiang Y Chen D Cai D Yi Y Jiang S. Effectiveness of remdesivir for the treatment of hospitalized COVID-19 persons: A network meta-analysis. J Med Virol (2021) 93(2):1171. doi: 10.1002/jmv.26443 32813283 PMC7461548

[62] Von Bernuth H Picard C Jin Z Pankla R Xiao H Ku CL. Pyogenic bacterial infections in humans with MyD88 deficiency. Science (2008) 321(5889):691–6. doi: 10.1126/science.1158298 PMC2688396 18669862

[63] Rao S Lee GM Razzaghi H Lorman V Mejias A Pajor NM. Clinical features and burden of post-acute sequelae of SARS-CoV-2 infection in children and adolescents: an exploratory EHR-based cohort study from the RECOVER program. medRxiv (2022). doi: 10.1101/2022.05.24.22275544

[64] Donnachie E Hapfelmeier A Linde K Tauscher M Gerlach R Greissel A. Incidence of post-COVID syndrome and associated symptoms in outpatient care in Bavaria, Germany: A retrospective cohort study using routinely collected claims data. BMJ Open (2022) 12(9):e064979. doi: 10.1136/bmjopen-2022-064979 PMC9511014 36137635

[65] Awwad AA Abd Elhay OMM Rabie MM Awad EA Kotb FM Maghraby HM. Impact of systemic diseases on olfactory function in COVID-19 infected patients. Int J Gen Med (2022) 15:5681–91. doi: 10.2147/IJGM.S355974 PMC9212789 35747780

[66] Kisiel MA Janols H Nordqvist T Bergquist J Hagfeldt S Malinovschi A. Predictors of post-COVID-19 and the impact of persistent symptoms in non-hospitalized patients 12 months after COVID-19, with a focus on work ability. Ups J Med Sci (2022) 127:10.48101/ujms.v127.8794. doi: 10.48101/ujms.v127.8794 PMC9383047 35991464

[67] Nguyen NN Hoang VT Dao TL Meddeb L Cortaredona S Lagier JC. Long-term persistence of olfactory and gustatory disorders in COVID-19 patients. Front Med (2022) 9. doi: 10.3389/fmed.2022.794550 PMC8915119 35280874

[68] Chudzik M Babicki M Mastalerz-Migas A Kapusta J. Persisting smell and taste disorders in patients who recovered from SARS-CoV-2 virus infection-data from the polish PoLoCOV-CVD study. Viruses (2022) 14(8):1763. doi: 10.3390/v14081763 36016385 PMC9416276

[69] Naeini AS Karimi-Galougahi M Raad N Ghorbani J Taraghi A Haseli S. Paranasal sinuses computed tomography findings in anosmia of COVID-19. Am J Otolaryngol (2020) 41(6):102636. doi: 10.1016/j.amjoto.2020.102636 32652405 PMC7831990

[70] Jarrott B Head R Pringle KG Lumbers ER Martin JH. “LONG COVID”–a hypothesis for understanding the biological basis and pharmacological treatment strategy. Pharmacol Res Perspect (2022) 10(1):e00911. doi: 10.1002/prp2.911 35029046 PMC8929332

[71] Drzymalla E Green RF Knuth M Khoury MJ Dotson WD Gundlapalli A. COVID-19-related health outcomes in people with primary immunodeficiency: A systematic review. Clin Immunol (2022) 243:109097. doi: 10.1016/j.clim.2022.109097 35973637 PMC9375253

[72] de Melo EGM Andrade RM de Abreu de Vasconcellos SJ dos Santos PL Tanajura DM Quintans-Júnior LJ. Association between chemosensory dysfunctions and inflammatory biomarkers in patients with SARS-CoV-2 infection: a systematic review and meta-analysis. Inflammopharmacology (2022) 30(6):2079–87. doi: 10.1007/s10787-022-01066-z PMC9467422 36097300

[73] Fragiel M Miró Ò Llorens P Jiménez S Piñera P Burillo G. Incidence, clinical, risk factors and outcomes of Guillain-Barré in covid-19. Ann Neurol (2021) 89(3):598–603. doi: 10.1002/ana.25987 33295021

[74] Rinaldi S. Coronavirus disease 2019 and the risk of Guillain–Barré syndrome. Ann Neurol (2021) 89(4):846. doi: 10.1002/ana.26011 33394537

[75] Filosto M Cotti Piccinelli S Gazzina S Foresti C Frigeni B Servalli MC. Guillain-Barré Syndrome and COVID-19: A 1-year observational multicenter study. Eur J Neurol (2022) 29(11):3358–67. doi: 10.1111/ene.15497 PMC9349567 35837806

[76] Hale M Netland J Chen Y Thouvenel CD Smith KN Rich LM. IgM antibodies derived from memory b cells are potent cross-variant neutralizers of SARS-CoV-2. J Exp Med (2022) 219(9):e20220849. doi: 10.1084/jem.20220849 35938988 PMC9365875

[77] Giardino G Milito C Lougaris V Punziano A Carrabba M Cinetto F. The impact of SARS-CoV-2 infection in patients with inborn errors of immunity: The experience of the Italian primary immunodeficiencies network (IPINet). J Clin Immunol (2022) 42(5):935–46. doi: 10.1007/s10875-022-01264-y PMC9020753 35445287

[78] Delmonte OM Bergerson JR E Burbelo PD Durkee-Shock JR Dobbs K Bosticardo M. Antibody responses to the SARS-CoV-2 vaccine in individuals with various inborn errors of immunity. J Allergy Clin Immunol (2021) 148(5):1192–7. doi: 10.1016/j.jaci.2021.08.016 PMC8418380 34492260

[79] Quinti I Lougaris V Milito C Cinetto F Pecoraro A Mezzaroma I. A possible role for b cells in COVID-19? lesson from patients with agammaglobulinemia. J Allergy Clin Immunol (2020) 146(1):211. doi: 10.1016/j.jaci.2020.04.013 32333914 PMC7175894

[80] Ponsford MJ Shillitoe BMJ Humphreys IR Gennery AR Jolles S. COVID-19 and X-linked agammaglobulinemia (XLA) - insights from a monogenic antibody deficiency. Curr Opin Allergy Clin Immunol (2021) 21(6):525–34. doi: 10.1097/ACI.0000000000000792 34596095

[81] Steiner S Schwarz T Corman VM Gebert L Kleinschmidt MC Wald A. SARS-CoV-2 T cell response in severe and fatal COVID-19 in primary antibody deficiency patients without specific humoral immunity. Front Immunol (2022) 13. doi: 10.3389/fimmu.2022.840126 PMC8960624 35359967

[82] Bartleson JM Radenkovic D Covarrubias AJ Furman D Winer DA Verdin E. SARS-CoV-2, COVID-19 and the aging immune system. Nat Aging (2021) 1(9):769–82. doi: 10.1038/s43587-021-00114-7 PMC8570568 34746804

[83] Unterman A Sumida TS Nouri N Yan X Zhao AY Gasque V. Single-cell multi-omics reveals dyssynchrony of the innate and adaptive immune system in progressive COVID-19. Nat Commun (2022) 13(1):440. doi: 10.1038/s41467-021-27716-4 35064122 PMC8782894

[84] Zhang Q Bastard P Karbuz A Gervais A Tayoun AA Aiuti A. Human genetic and immunological determinants of critical COVID-19 pneumonia. Nature (2022) 603(7902):587–98. doi: 10.1038/s41586-022-04447-0 PMC8957595 35090163

[85] Soleimanian S Alyasin S Sepahi N Ghahramani Z Kanannejad Z Yaghobi R. An update on protective effectiveness of immune responses after recovery from COVID-19. Front Immunol (2022) 13:884879. doi: 10.3389/fimmu.2022.884879 35669767 PMC9163347

[86] Pierce CA Preston-Hurlburt P Dai Y Aschner CB Cheshenko N Galen B. Immune responses to SARS-CoV-2 infection in hospitalized pediatric and adult patients. Sci Transl Med 12(564):eabd5487. doi: 10.1126/scitranslmed.abd5487 PMC7658796 32958614

[87] Kinoshita H Durkee-Shock J Jensen-Wachspress M Kankate VV Lang H Lazarski CA. Robust antibody and T cell responses to SARS-CoV-2 in patients with antibody deficiency. J Clin Immunol (2021) 41(6):1146–53. doi: 10.1007/s10875-021-01046-y PMC8117127 33983545

[88] Jones JM Faruqi AJ Sullivan JK Calabrese C Calabrese LH. COVID-19 outcomes in patients undergoing b cell depletion therapy and those with humoral immunodeficiency states: A scoping review. Pathog Immun (2021) 6(1):76. doi: 10.20411/pai.v6i1.435 34056149 PMC8150936

[89] Gupta S Agrawal S Sandoval A Su H Tran M Demirdag Y. SARS-CoV-2-Specific and functional cytotoxic CD8 cells in primary antibody deficiency: Natural infection and response to vaccine. J Clin Immunol (2022) 42(5):914–22. doi: 10.1007/s10875-022-01256-y PMC8976534 35366743

[90] Shields AM Tadros S Al-Hakim A Nell JM Lin MMN Chan M. Impact of vaccination on hospitalization and mortality from COVID-19 in patients with primary and secondary immunodeficiency: The united kingdom experience. Front Immunol 13:984376. doi: 10.3389/fimmu.2022.984376 36211396 PMC9539662

