# Supplementary material for: Acute and long-term immune responses to SARS-CoV-2 infection in unvaccinated children and young adults with inborn errors of immunity
Source: Front Immunol. 2023 Jan 20;14:1084630. doi: 10.3389/fimmu.2023.1084630 (PMC9896004; doi:10.3389/fimmu.2023.1084630)
Supplement: Supplementary Table 1 — Patients with IEI baseline clinical characteristics and SARS-CoV-2 infection description. [file Table_1.pdf]

Supplementary table 1. Patients with IEI baseline clinical characteristics and SARS-CoV-2 infection description.

Patient	Gender	Age (years)	IEI[a]	COVID-19 risk factors	Baseline treatment			COVID-19				Publication
					IgRT	Antibiotic	Immunosupressant	Severity	Symptoms	Treatment	Outcome	
**P1**	M	9	MHCII def. (*RFXANK*)	No	Yes	Cotrimoxazole, posaconazole	Systemic corticosteroids	Moderate	Fever, non-hypoxemic pneumonia	Remdesivir, empiric antibiotic	Recovered	No
**P2**	M	9	MHCII def. (*RFXANK*)	No	Yes	Cotrimoxazole, posaconazole	Systemic corticosteroids	Mild	Asymptomatic	Remdesivir	Recovered	No
**P3**	F	17	$CD4^+$ idiopathic lymphopenia	No	No	No	No	Mild	Low-grade fever, myalgia, asthenia, smell and taste loss	No	Dysgeusia, dysosmia (for 2 years)	No
**P4**	F	12	PGM3 def.	No	No	No	Dupilumab	Moderate	Cough, low-grade fever Ground-glass opacity on chest scan	No	Recovered	Deyà-Martínez *et al.*, 2021; García-García *et al.*, 2021
**P5**	M	19	A-T (*ATM*)	No	Yes	No	No	Mild	Fever No pneumonia (chest X-ray)	Empiric antibiotic	Recovered	
**P6**	F	13	Jacobsen sd (11q del.)	No	No	No	No	Mild	Cough	No	Recovered	
**P7**	M	17	CATCH22 (22q11del,)	No	No	No	No	Mild	Asymptomatic	No	Recovered	
**P8**	F	16	Down sd. (47,XX+21)	Down syndrome, mitral regurgitation	No	No	No	Mild	Asymptomatic	No	Recovered	Deyà-Martínez *et al.*, 2021
**P9**	M	10	Jacobsen sd. (11q del.)	No	No	No	No	Mild	Asymptomatic	No	Recovered	
**P10**	M	5	CATCH22 (22q11 del.)	Cyanotic congenital heart disease	No	No	No	Mild	Fever	No	Recovered	
**P11**	M	10	CATCH22 (22q11del.)	No	No	No	No	Mild	Asymptomatic	No	Recovered	No

Patient	Gender	Age (years)	IEI[a]	COVID-19 risk factors	Baseline treatment			COVID-19				Publication
					IgRT	Antibiotic	Immunosupressant	Severity	Symptoms	Treatment	Outcome	
**P12**	F	4	Charge sd. (*CHD7)*	Mild SAHS	No	No	No	Mild	Asymptomatic		Recovered	No
**P13**	M	13	Kabuki sd. (*KMDT2)*	No	No	No	No	Mild	Odynophagia, fever		Recovered	No
**P14**	M	9	THE sd. (*TTC37*)	No	Yes	No	No	Mild	Cough, fever, runny nose, diarrhea	No	Recovered	No
**P15**	F	14	Sotos sd (*NSD1*)	No	No	No	No	Mild	Headache, asthenia	No	Recovered	No
**P16**	M	8	Bruton sd. (*BTK*)	No	Yes	No	No	Mild	Asymptomatic	No	Recovered	Deyà-Martínez *et al*., 2021
**P17**	F	17	CVID	No	No	No	No	Mild	Asymptomatic	No	Recovered	
**P18**	M	16	CVID	No	No	No	No	Mild	Low-grade fever, odynophagia	No	Recovered	No
**P19**	F	14	CVID	No	No	No	No	Mild	Asymptomatic	No	Recovered	Deyà-Martínez *et al*., 2021
**P20**	M	18	ALPS	No	No	No	No	Mild	Odynophagia, retroocular pain, runny nose, loss of smell and taste	No	Recovered	
**P21**	M	15	STAT1 GOF	No	No	Fluconazole	No	Mild	Asymptomatic	No	Guillain-Barré[b]	
**P22**	F	7	STAT1 GOF	No	Yes	Fluconazole, acyclovir	Ruxolitinib	Mild	Asymptomatic	No	Recovered	No
**P23**	M	23	CGD (*NCF1*)	No	No	Cotrimoxazole, itraconazole	Corticosteroids	Mild	Asymptomatic	No	Recovered	Von Bernuth *et* al., 2018 Deyà-Martínez *et al*., 2021
**P24**	M	19	MyD88 def.	Obesity	No	No	No	Severe	Fever, cough Hypoxemic pneumonia (no MV)	Corticoids Remdesivir Antibiotic Heparin	Recovered	
**P25**	F	17	MyD88 def.	No	No	No	No	Moderate	Fever, cough Non-hypoxemic pneumonia	Corticoids Remdesivir Antibiotic	Recovered	

[a]The genetic defect is described if known; otherwise, we only provide the immunodeficiency according to the ESID clinical criteria classification (2019)(46) [b]Guillain-Barré after 9 months of testing positive for SARS-CoV-2, with no other infectious trigger detected in the routine screening. ALPS: autoimmune lymphoproliferative syndrome; A-T: ataxia-telangiectasia; CGD: chronic granulomatous disease; def.: deficiency; GOF: gain-of-function; IEI: Inborn errors of immunity; MHCII: major histocompatibility complex II; MMF: mycophenolate mofetil; MV: mechanical ventilation; SAHS: sleep apnea-hypopnea syndrome; sd.: syndrome; THE: trichohepatoenteric; 22q11del: 22q11 deletion.
