# Supplementary material for: Acute and long-term immune responses to SARS-CoV-2 infection in unvaccinated children and young adults with inborn errors of immunity
Source: Front Immunol. 2023 Jan 20;14:1084630. doi: 10.3389/fimmu.2023.1084630 (PMC9896004; doi:10.3389/fimmu.2023.1084630)
Supplement: Supplementary Table 2 — Comparison of SARS-CoV-2 infection in IEI and non-IEI patients. [file Table_2.pdf]

Supplementary table 2. Comparison of SARS-CoV-2 infection in IEI and non-IEI patients.

	IEI (n=25)	Non-IEI (n=17)	*p*-value
**Age (years)[a]**	14.3 (4.5-22.8)	15.3 (5.4-20.0)	0.5
**Male**	15/25 (60.0%)	6/17 (35.3%)	0.1
**COVID-19 pneumonia**	4/25 (16.0%)	1/17 (5.9%)	0.3
**Hypoxemic pneumonia**	1/25 (4.0%)	1/17 (5.9%)	0.6
**Hospitalization**	4/25 (16.0%)	1/17 (5.9%)	0.3
**SARS-CoV-2 specific management**	4/25 (16.0%)	1/17 (5.9%)	0.3
**Symptoms[b]**	13/25 (52.0%)	13/17 (76.5%)	0.1
**Fever**	7/25 (28.0%)	5/17 (29.4%)	0.6
**Cough**	5/25 (20.0%)	1/17 (5.9%)	0.2
**Low-grade fever**	3/25 (12.0%)	0/17 (0%)	0.2
**Odynophagia**	3/25 (12.0%)	1/17 (5.9%)	0.5
**Runny nose**	2/25 (8.0%)	4/17 (23.5%)	0.2
**Asthenia**	2/25 (8.0%)	2/17 (11.8%)	0.5
**Smell and taste loss**	2/25 (8.0%)	0/17 (0%)	0.4
**Headache**	1/25 (4.0%)	7/17 (41.2%)	**0.004**<br>(OR 0.06; 95% CI 0.006-0.5)
**Diarrhea**	1/25 (4.0%)	1/17 (5.9%)	0.6
**Myalgia**	1/25 (4.0%)	1/17 (5.9%)	0.6
**Ocular pain**	1/25 (4.0%)	0/17(0%)	0.6
**Smell and taste loss**	2/25 (8.0%)	0/17(0%)	0.3

[a]Age is expressed as median (min-max). [b]Presence of one or more symptoms
